# Supplementary material for: Spatiotemporal Patterns of Aquatic Product Risks in China Based on Entropy-Weighted TOPSIS
Source: Foods. 2025 Dec 11;14(24):4263. doi: 10.3390/foods14244263 (PMC12733135; doi:10.3390/foods14244263)
Supplement: Supplementary file 1 [file foods-14-04263-s001.zip › foods-4009929-supplementary.pdf]

Supplementary Materials:

Table S1. Risk severity of hazardous substances in food assignment table

Risk Indicators	Classification	Hazard degree score
Health guidance values (ADI, TDI, and others)	>100	1
	(10-100]	2
	(1-10]	3
	(0.1-1]	4
	≤0.1	5
Lethal dose 50 (LD50)	>5000	1
	(500-5000]	2
	(50-500]	3
	(1-50]	4
	≤1	5
Carcinogenicity	Non-carcinogens (Group 4)	1
	Not yet classifiable (Group 3)	2
	Possibly carcinogenic to humans (Group 2B)	3
	Probably carcinogenic to humans (Group 2A)	4
	Definitely carcinogenic to humans (Group 1)	5

Table S2. The non-compliance rates of various risk substances across different provinces

Provinces /Hazardous substance	Sulfur dioxide	Cadmium	Enrofloxacin	Methylmercury	Furazolidone metabolites	Nitrofurantoin metabolites	Metronidazole	Chloramphenicol
Beijing	0.00	2.67	1.69	0.00	0.00	0.00	0.00	0.08
Tianjin	12.50	2.86	0.68	0.00	0.00	0.00	0.00	0.10
Hebei	0.00	12.73	0.88	0.00	0.00	0.00	0.15	0.11
Shanxi	0.00	0.16	2.38	0.00	0.00	0.00	0.00	0.00
Inner Mongolia	0.00	4.87	1.07	0.00	0.00	0.23	0.00	0.00
Liaoning	2.13	6.05	1.49	0.00	0.00	0.00	0.00	0.49
Jilin	0.43	9.01	0.93	0.00	0.00	0.00	0.00	0.00
Heilongjiang	2.86	2.67	1.30	0.00	0.00	0.00	0.00	0.00
Shanghai	0.00	1.69	4.67	0.00	0.00	0.00	0.00	0.04
JIANGSU	0.00	3.30	5.59	0.00	0.00	0.00	0.00	0.05
Zhejiang	0.00	0.69	12.02	0.00	0.00	0.00	0.14	0.15
Anhui	0.00	1.53	6.72	0.00	0.00	0.00	0.10	0.00
Fujian	0.00	3.19	3.71	0.00	0.00	0.00	0.00	0.27
Jiangxi	0.00	6.91	13.14	0.00	0.11	0.08	0.00	0.07
Shandong	8.06	3.29	0.89	0.00	0.00	0.00	0.10	0.07
Henan	3.03	2.22	1.43	0.00	0.00	0.00	0.00	0.04
Hubei	0.00	0.11	5.05	0.00	0.00	0.00	0.00	0.22
Hunan	0.00	2.27	3.86	0.00	0.00	0.00	0.00	0.00
Hunan	0.00	4.55	2.49	0.00	0.00	0.00	0.00	1.05
Guangxi	0.00	0.24	2.40	0.00	0.00	0.00	0.00	1.73
Hainan	0.00	0.23	2.78	0.00	0.00	0.00	0.00	1.15
Chongqing	0.00	0.00	12.20	0.00	0.00	0.00	0.26	0.18
Sichuan	0.00	0.00	6.05	0.00	0.00	0.00	0.00	0.20
Guizhou	0.00	0.42	3.33	0.00	0.00	0.00	0.00	0.00
Yunnan	9.09	0.00	5.30	0.00	0.00	0.00	0.00	0.11
Tibet	0.00	0.00	2.65	0.00	0.00	0.00	0.00	0.00
Shannxi	0.00	0.70	2.45	0.00	0.00	0.00	0.70	0.00

Gansu	0.00	0.65	1.55	0.00	0.00	0.00	0.32	0.00
Qinghai	7.41	0.36	1.90	0.00	0.00	0.00	0.00	0.00
Ningxia	0.00	0.43	0.17	0.00	0.00	0.00	0.00	0.00
Xinjiang	0.00	0.32	1.63	0.00	0.00	0.00	0.00	0.00

Table S3. The non-compliance rates of various risk substances across different provinces.

Provinces/Hazardous substance	Malachite green	Diazepam	Furazolidone metabolites	Nitrofurazone metabolites	Sodium pentachlorophenolate	Ofloxacin	Total volatile basic nitrogen	Sarafloxacin
Beijing	1.04	2.15	0.18	0.00	0.93	0.00	0.50	0.00
Tianjin	0.35	1.10	0.00	0.00	0.00	0.00	0.72	0.00
Hebei	0.35	2.43	0.18	0.07	0.15	0.23	0.00	0.00
Shanxi	0.14	0.44	0.07	0.00	0.00	1.49	0.00	0.00
Inner Mongolia	0.29	0.00	0.00	0.00	0.00	0.75	1.24	0.00
Liaoning	0.12	0.85	0.00	0.11	0.00	0.00	0.00	0.00
Jilin	0.02	1.29	0.00	0.00	0.05	0.00	0.46	0.00
Heilongjiang	0.04	0.25	0.00	0.00	0.00	0.00	0.00	0.00
Shanghai	0.25	0.15	0.31	0.44	0.00	0.03	0.00	0.00
JIANGSU	0.29	0.21	0.23	0.03	0.04	0.00	0.15	0.00
Zhejiang	0.10	0.71	1.54	0.91	0.43	0.14	0.00	0.00
Anhui	0.36	0.52	0.00	0.00	0.00	0.27	0.38	0.00
Fujian	0.06	0.00	1.16	0.04	0.04	0.11	0.17	0.00
Jiangxi	0.34	0.00	0.09	0.72	0.00	0.41	0.00	0.00
Shandong	0.09	1.63	0.03	0.15	0.23	0.00	0.05	0.00
Henan	0.17	0.61	0.16	0.06	0.03	0.07	0.00	0.00
Hubei	0.27	0.25	0.11	0.00	0.10	0.33	0.00	0.00
Hunan	0.28	0.05	0.18	0.08	0.00	0.00	1.27	0.00
Hunan	0.53	0.00	0.69	0.34	0.00	0.00	0.00	0.00
Guangxi	1.25	0.36	0.54	0.04	0.00	0.00	0.00	0.00
Hainan	0.16	0.00	0.69	0.26	0.05	0.24	0.00	0.00
Chongqing	0.40	0.71	0.77	1.08	0.16	0.17	0.00	0.00
Sichuan	0.36	2.76	0.38	0.54	0.68	0.00	2.94	0.00
Guizhou	0.16	0.15	0.25	0.10	0.13	0.00	0.00	0.00
Yunnan	1.42	0.87	0.06	0.00	0.20	0.38	0.00	0.00
Tibet	0.00	0.00	0.00	0.00	0.00	0.00	0.00	0.00
Shannxi	0.69	0.95	0.27	0.00	0.00	5.48	1.12	0.00
Gansu	0.60	0.00	0.12	0.00	0.00	4.52	0.00	0.00
Qinghai	0.42	0.00	0.14	0.00	0.30	2.28	0.00	0.00
Ningxia	0.09	0.87	0.00	0.00	0.00	0.49	0.00	0.00
Xinjiang	0.06	1.56	0.06	0.00	0.00	1.01	0.23	0.00

Table S4. Distribution of samples across chinese provinces.

Provinces	Qualified Quantity	Number of Nonconforming Items
Shanghai	80841	481
Henan	61557	203
Beijing	59690	369
Shandong	57458	218
Jiangsu	51291	347
Jilin	49866	462
Hunan	46157	210
Guangxi	44099	211
Guangdong	44031	333
Zhejiang	43361	720
Hainan	41782	213
Fujian	40809	306
Guizhou	37578	137
Jiangxi	35868	530
Heilongjiang	32909	96
Sichuan	32697	248
Chongqing	32588	494
Anhui	29403	198
Liaoning	26989	168
Tianjin	24716	79
Hubei	24080	120
Yunnan	21076	142
Hebei	20265	212
Shanxi	18287	43
Ningxia	15544	13
Shaanxi	15291	108
Inner Mongolia	12726	53
Xinjiang	23354	54
Gansu	9293	21
Qinghai	9292	24
Tibet	1414	3
